# Early Outcomes of Percutaneous Pulmonary Valve Implantation with Pulsta and Melody Valves: The First Report from Korea

**DOI:** 10.3390/jcm9092769

**Published:** 2020-08-26

**Authors:** Ah Young Kim, Jo Won Jung, Se Yong Jung, Jae Il Shin, Lucy Youngmin Eun, Nam Kyun Kim, Jae Young Choi

**Affiliations:** 1 Division of Pediatric Cardiology, Center for Congenital Heart Disease, Severance Cardiovascular Hospital, Yonsei University College of Medicine, Seoul 03722, Korea; dalkiay@yuhs.ac (A.Y.K.); jwjung@yuhs.ac (J.W.J.); jung811111@yuhs.ac (S.Y.J.); SHINJI@yuhs.ac (J.I.S.); LUCYEUN@yuhs.ac (L.Y.E.); 2 Department of Pediatrics, Yonsei University College of Medicine, Seoul 03722, Korea; 3 Department of Pediatrics, Emory University, Atlanta, GA 30322, USA; nam.kyun.kim@emory.edu

**Keywords:** heart diseases, heart valve prosthesis, pulmonary heart disease, pulmonary valve, pediatrics

## Abstract

Percutaneous pulmonary valve implantation (PPVI) is used to treat pulmonary stenosis (PS) or pulmonary regurgitation (PR). We described our experience with PPVI, specifically valve-in-valve transcatheter pulmonary valve replacement using the Melody valve and novel self-expandable systems using the Pulsta valve. We reviewed data from 42 patients undergoing PPVI. Twenty-nine patients had Melody valves in mostly bioprosthetic valves, valved conduits, and homografts in the pulmonary position. Following Melody valve implantation, the peak right ventricle-to-pulmonary artery gradient decreased from 51.3 ± 11.5 to 16.7 ± 3.3 mmHg and right ventricular systolic pressure fell from 70.0 ± 16.8 to 41.3 ± 17.8 mmHg. Thirteen patients with native right ventricular outflow tract (RVOT) lesions and homograft underwent PPVI with the new self-expandable Pulsta valve—a nitinol wire stent mounted with a trileaflet porcine pericardial valve. Following Pulsta valve implantation, cardiac magnetic resonance imaging showed a decreased PR fraction and that the right ventricular end-diastolic volume index decreased from 166.1 ± 11.9 to 123.6 ± 12.4 mL/m^2^. There were no mortality, severe procedural morbidity, or valve-related complications. At the mean 14.2 month (4–57 months) follow-up, no patients had more than mild PR. PPVI using Melody and Pulsta valves was first shown to provide excellent early outcomes without serious adverse event in most patients with RVOT dysfunction in Korea.

## 1. Introduction

Patients with either congenital or acquired heart diseases involving dysfunction of the right ventricular outflow tract (RVOT) require implantation of a prosthetic valve, with or without a conduit, between the right ventricle (RV) and the pulmonary artery (PA). This implantation is required for significant pulmonary stenosis or pulmonary regurgitation (PR) and ventricular failure as well [1]. However, given that the inevitable degeneration of the artificial tissue of the prosthetic valves and conduits results in clinically significant pulmonary regurgitation or stenosis, repetitive and multiple surgical interventions turn out to be mandatory. Overall, this leads to greater scarring of the cardiac and vascular tissues and an increased surgical risk. Furthermore, open-heart surgery is associated with considerable psychological strain on the patient, as well as with morbidity and lengthy recovery times [2].

Percutaneous pulmonary valve implantation (PPVI) using Melody (Medtronic, Minneapolis, MN, USA) and Edwards SAPIEN (Edwards Lifesciences, Irvine, CA, USA) valves has been used to replace surgical pulmonary valve replacement and extend the lifespan of existing RVOT revisions to delay the need for repeated surgical interventions [3]. This technique is considered the most appropriate first-line approach to pulmonary valve replacement in selected patients [4]. Since its first implementation in 2000 by Bonhoeffer et al. [3] and subsequent approval by the Food and Drug Administration in 2010, PPVI using the Melody and Edwards SAPIEN valves has been performed in more than 13,000 patients with postoperative RVOT dysfunction worldwide. Indeed, surgically implanted RV-to-PA valves and conduits are being replaced by balloon-expandable PPVIs, which showed excellent long-term outcome by several reports [5,6,7,8,9,10]. However, recent advances such as valve-in-valve replacement within dysfunctional bioprosthetic valves (BPVs) and smaller conduits in smaller patients (<30 kg), or recognized factors for a better outcome such as the routine use of pre-stenting and the avoidance of high post-interventional gradients across RVOT, are not uniformly applied to existing studies.

Concurrently the new larger valve of a self-expandable nature has been suggested as the next generation of percutaneous valves in majority of patulous native RVOT lesions [11,12]. The Pulsta valve (TaeWoong Medical Co, Gyeonggi-do, South Korea) is a novel, self-expandable valve with flared ends that adapts well to a larger native RVOT. It uses a relatively low-profile delivery catheter with a knitted nitinol wire backbone and trileaflets made from treated porcine pericardial tissue [13]. Since their efficacy and safety were reported in a study published in 2018, cases of Pulsta valve implantation have increased in South Korea [14].

In 2015, PPVI with Melody valves was performed for the first time in Korea in three patients at our institution. Thereafter, improvements were observed in both the procedure and the perception of the device by domestic congenital cardiologists. Recently, the use of both the Melody and Pulsta valves has been approved by the Korean Food and Drug Administration. This is the first report discussing the use of both valves to treat patients with PPVI. This study aimed to evaluate the early procedural and clinical outcomes of PPVI using the Melody and Pulsta valves.

## 2. Experimental Section

### 2.1. Study Population

A total of 42 patients who underwent PPVI in a single tertiary referral center from August 2015 to March 2020 were retrospectively enrolled in the current study. Criteria for a PPVI included the following: moderate or substantial pulmonary regurgitation, indexed RV end-diastolic volume > 150 mL/m^2^, indexed RV end-systolic volume > 80 mL/m^2^, or RV dysfunction (RV ejection fraction < 47%) as observed on cardiac magnetic resonance image (MRI), and/or pulmonary stenosis (with a mean RVOT gradient ≥ 35 mmHg) as measured by transthoracic echocardiography (TTE) [1,15,16]. Additional clinical features were exercise intolerance, symptoms and signs of heart failure, syncope, and/or significant arrhythmia. Finally, the patient’s anatomy was evaluated for the implantation of either the Melody or Pulsta valve through TTE, cardiac computed tomography (CT), cardiac MRI, and cardiac angiography.

### 2.2. The Valves

#### 2.2.1. The Melody Valve

The Melody valve is a transcatheter pulmonary valve (TPV) consisting of a bovine jugular vein sutured inside a platinum iridium stent. Currently, two valve sizes are available, namely the TPV 20 and 22. While the TPV 20 uses a 16 mm bovine jugular vein and is intended for 20 mm diameter implantations, the TPV 22 uses an 18 mm bovine jugular vein intended for 22 mm diameter implantations. Although the unexpanded valve heights are 30 and 28 mm for the TPV 20 and 22, respectively, they are both deployed using the Medtronic Ensemble delivery system, which is a 22 F delivery system that uses balloon-in-balloon technology. Finally, this ensemble is available in three sizes, i.e., 18, 20, and 22 mm [3,17].

#### 2.2.2. The Pulsta Valve

The Pulsta valve is made of a knitted double-strand nitinol wire, and its stent wall is covered with trileaflets made from treated porcine pericardial tissue. The valve diameter ranges from 18 to 32 mm (with increments of 2 mm). Both ends of the valve are flared, rendering the valve 4 mm wider than its outer diameter at its ends to ensure stable valve adaptation to various RVOT geometries. Therefore, the total length of the valve varies between 28 and 38 mm, according to its outer diameter. While the diameter of the outer sheath in the valve loading zone is 18 F (5.9 mm), that of the catheter shaft is 12 F. Also, hooking the proximal end of the nitinol wires to the hook block simply allows for both controlled deployment and subsequent good positioning of the valve at the target area [13,14].

### 2.3. Clinical Assessment

For the monitoring and assessment of patients’ outcome, we performed regular follow-up at 1 month, 3–6 months, 12 months, and annual visit after implantation. The primary outcome measures were the feasibility and safety of valve implantation, determined in patients whose TPV had been implanted for > 24 h; the outcome measure was a composite of the following evaluations: physical assessments, exercise testing, cardiac MRI, and transthoracic echocardiography. The following factors were measured to determine the hemodynamic and functional outcomes of implantation: RV and PA systolic pressures (measured by cardiac catheterization), peak systolic gradient over the RVOT (measured by Doppler recording on transthoracic echocardiography), degree of PR and TR (measured by transthoracic echocardiography and cardiac MRI), indexed right ventricular end-diastolic volume (RVEDVi) and RV ejection fraction (measured by cardiac MRI). In addition, follow-up data included the changes in the New York Heart Association (NYHA) class, peak oxygen consumption (VO_2_), anaerobic threshold (AT), device function, and evidence of structural valve deterioration, including stent fracture. Safety outcomes were specifically defined as a result of device malfunction as well as coronary compression, neurologic impairment, conduit/RVOT rupture, stent dislocation, bleeding complications, need for surgical correction, endocarditis of the implanted valve, and any cardiovascular events, including death, myocardial infarction, coronary compression, pulmonary embolism, and stroke/transient ischemic attack.

### 2.4. Statistical Analysis

Frequency distributions are provided for categorical variables, and means with standard deviations and medians with ranges are given for numerical variables. Statistical analyses were performed using SPSS Statistics version 23.0 for Windows (SPSS Inc., Chicago, IL, USA). Paired t tests were used to analyze changes in continuous variables between baseline and follow-up variables. Finally, the Wilcoxon signed-rank test was used to compare variables between the baseline and follow-up measurements. *p*-values less than 0.05 were considered statistically significant.

### 2.5. Ethics Statement

This study was approved by the Yonsei University College of Medicine Institutional Review Board and the Research Ethics Committee of Severance Hospital (study approval number: 4-2020-0210). All research was performed in accordance with relevant guidelines and regulations. The requirement for written informed consent was waived by the Institutional Review Board due to the retrospective study design.

## 3. Results

### 3.1. Patient Characteristics

The majority of patients were male (71.4%), with a mean age of 27.2 ± 13.5 years (median: 23 years old, range: 9.5–75 years). Baseline patient characteristics prior to the procedure are described in Table 1. Among the 42 patients, twelve presented with a native RVOT anatomy and 1 with a pulmonary homograft 26 mm prior to PPVI (31.0%) with the Pulsta valve, 28 demonstrated stented bioprosthetic valves and conduits for the most common RVOT anatomy findings prior to implantation of the Melody valve (e.g., Carpentier-Edwards, Hancock, Saint Jude Epic; 28/42, 66.7%), and one case of pulmonary homograft was also observed. Moreover, 29 patients showed an above-moderate RVOT obstruction, which corresponded to the eligibility criteria for the implantation procedure. The thirteen patients who opted for the Pulsta valve presented with severe PR, with a mean PR fraction derived by cardiac MRI of 46.7% ± 8.0% (range: 38.0–59.0%). Meanwhile, the patients using the Melody valves showed a mean PR fraction of 17.1% ± 13.6% (range: 1.2–40.2%) better than Pulsta valve group (*p* = 0.02). The mean indexed RV end-diastolic volume was 166.1 ± 11.9 mL/m^2^, as observed with cardiac MRI in Pulsta valve patients, significantly larger than that in patients with the Melody valve (137.5 ± 31.1, *p* = 0.04). Furthermore, all patients showed mild-to-moderate exercise intolerance during cardiopulmonary exercise assessments, with a mean peak oxygen consumption of 28.1 ± 4.7 mL/kg/min (range: 19.4–36.1 mL/kg/min). Finally, most patients belonged to either NYHA class II (24/42, 57.1%) or III (11/42, 26.2%) at baseline.

### 3.2. Procedural Characteristics

Almost all PPVI procedures were conducted via the transfemoral approach with general anesthesia in 97.6% of subjects (Table 2), except one case of inferior vena cava interruption via right internal jugular vein access. The largest 22 mm delivery system was used for 29 patients who underwent Melody valve implantation. Specifically, while the minimal RVOT diameter was 20.5 ± 2.9 mm (range: 12–23 mm) prior to dilation, it increased to 22.1 ± 0.6 mm (range: 21.5–23 mm) thereafter. In 57.1% of patients (24/42), the Melody valve was placed into a previously implanted bioprosthesis (i.e., Carpentier-Edwards Perimount in eleven patients, Saint Jude Biocor and Epic in eleven, Hancock II in one, Shelhigh in one, and Prima Plus in one). In eight subjects, frame fracture was attempted after the achievement of a larger inner diameter and improved outcome. The RVOT was pre-stented in ten (23.8%) patients, either prior to the procedure (one subject) or on the day of the implantation (nine subjects). The Palmaz 4014 bare metal stent was chosen for most cases; however, the Cheatham Platinum (CP) covered stent was used in two case due to severe supravalvular stenosis and extensive calcification. In addition, valves with diameters of 26, 28, 30, and 32 mm were successively implanted in one, three, two, and seven patients, respectively, as Pulsta valves. Valve sizes were decided according to the measurement of the largest diameter of the target PA’s proximal, narrowest, and distal parts, as well as its length assessed via cardiac CT, MRI, and echocardiography and balloon sizing of RVOT simultaneously with RV angiography. The mean total procedural time was 95.3 ± 35.8 min, with a mean fluoroscopy time of 35.2 ± 10.1 min.

### 3.3. Periprocedural Outcomes and Complications during Follow-Up

Procedural success was defined as the implantation of a single valve in the intended location and was reported to be 100.0% in the current study (Table 3). No infective endocarditis and valve thrombosis were seen during the follow-up (mean: 14.2 months; range: 4–57 months). However, transient periprocedural complications were observed in one patient as a stent dislocation, which was successfully managed by repositioning without surgery and with the implantation of a Melody valve. Nevertheless, no further procedural complications and mortality were reported, except for one case of mortality due to unrelated complications that occurred during the follow-up period. Although PPVI was completed without any adverse events, the patient’s underlying life-threatening comorbidities and chronic renal failure were aggravated after the procedure; similarly, disseminated intravascular coagulation was not well tolerated. Finally, even though one case of Melody valve stent fracture was diagnosed during the evaluation conducted at 46 months after the implantation, this was a type I fracture [18], which did not require reintervention. We used pre-closure of large-sized sheath venous access sites using single or double suture-mediated Perclose device in over 90% patients. Although the diameter of the outer sheath in the valve loading zone is 22 Fr of Ensemble delivery system in Melody valve, which is larger than the 18 F diameter in Pulsta valve system, all patients showed no significant vascular complication or difficulty related to venous access.

### 3.4. Functional Outcomes

**Figure 1:**
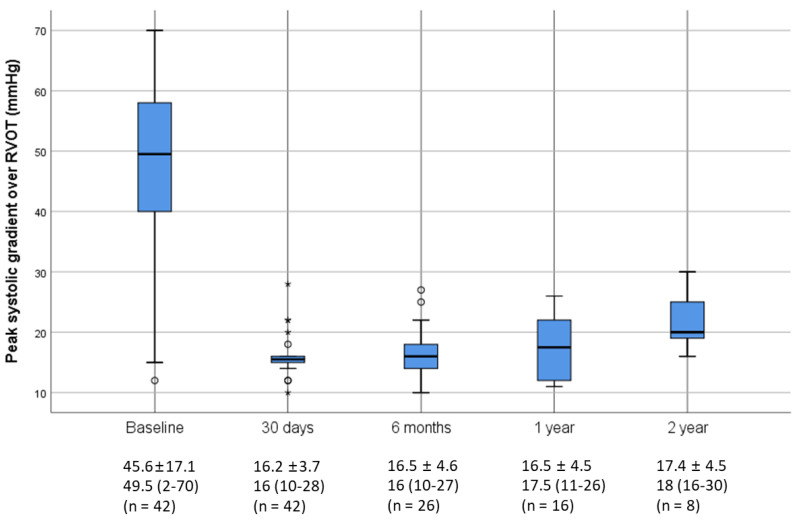
Peak pressure gradients between the right ventricle and the pulmonary artery before and after valve implantation by transthoracic echocardiography. There was no significant pulmonary valve stenosis over time. Values are presented as either the mean ± standard deviation or median (interquartile range). The box plot uses the median and the lower and upper quartiles (defined as the 25th and 75th percentiles). Extreme outliers are marked with an asterisk (*) on the boxplot. Mild outliers are data points that are more extreme than interquartile range, marked using a circle (o). RVOT, right ventricular outflow tract.

**Figure 2:**
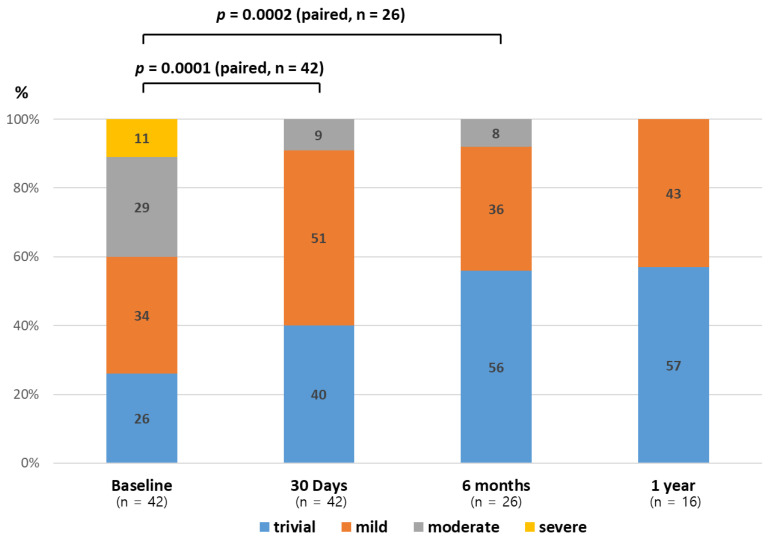
Changes in tricuspid valve regurgitation over time: before and after pulmonary valve implantation, as seen on transthoracic echocardiography. The proportion of greater than moderate tricuspid valve regurgitation decreased after pulmonary valve implantation.

**Figure 3:**
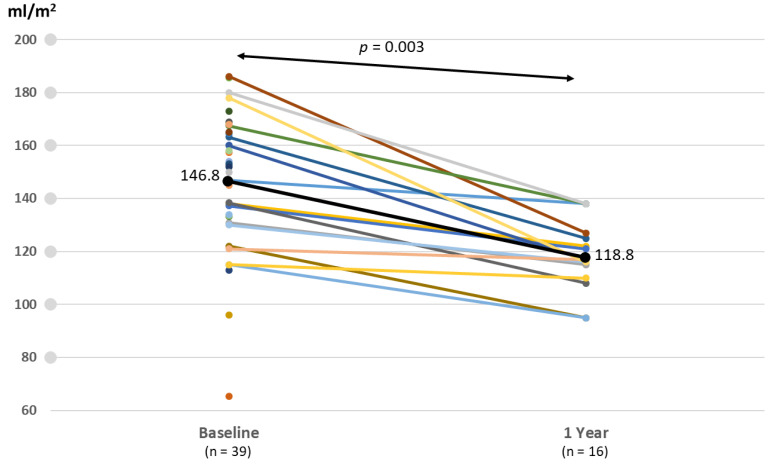
Changes in right ventricular volume before and 1 year after valve implantation. The mean indexed RV (right ventricle) end-diastolic volume index was significantly decreased after valve implantation, as seen on a cardiac MRI (magnetic resonance image).

**Figure 4:**
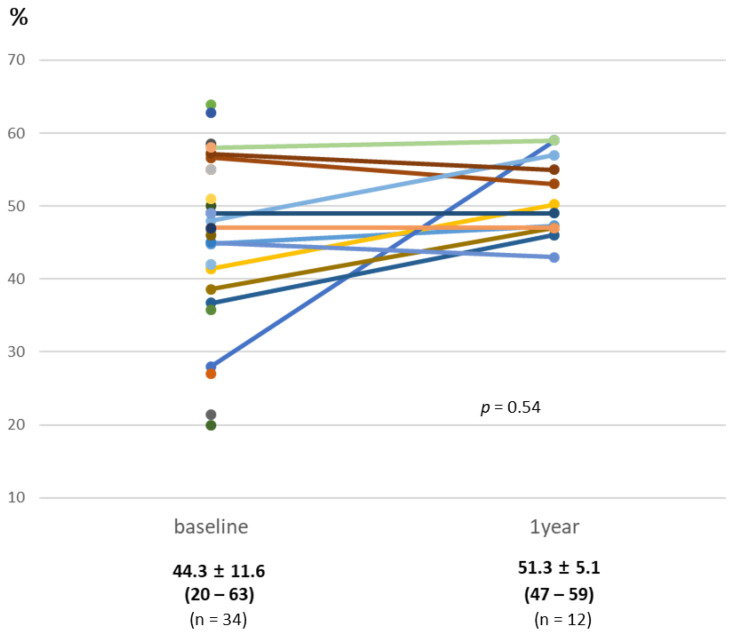
RV (right ventricle) ejection fraction (%) over time, as seen with cardiac MRI (magnetic resonance image). Values are presented as the mean ± standard deviation (range).

**Figure 5:**
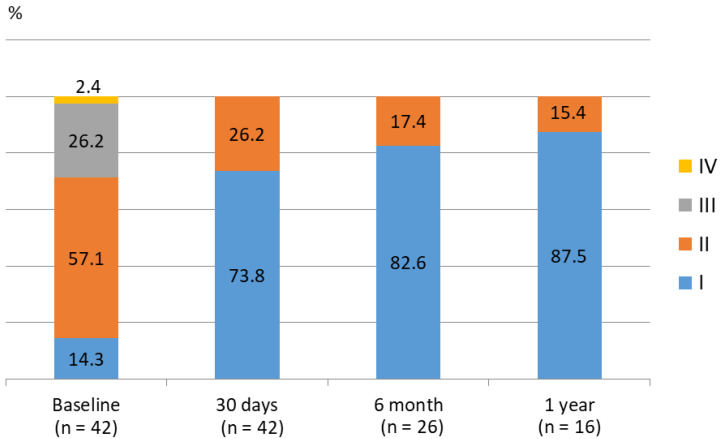
NYHA (New York Heart Association) functional class before and after PPVI (percutaneous pulmonary valve implantation). The NYHA functional class of patients improved to class I in all patients over time.

An improvement in hemodynamics and function was seen in patients following PPVI (Table 4). By post-procedural day 30, the median peak systolic gradient over the RVOT measured by Doppler recording on transthoracic echocardiography had fallen from 49.5 (preprocedural median) to 16 mmHg and slightly increased at 12 months without statistical significance (Figure 1). Specifically, these improvements were more noticeable in the Melody valve group. A similar trend was observed in right ventricular systolic pressure following Melody valve implantation. Furthermore, while 25 patients (60%) had moderate-to-severe PR at baseline, no subjects displayed PR after PPVI. Quantitative analysis of PR conducted with follow-up cardiac MRI also showed improvement in both the Melody and the Pulsta valve groups (3.8 ± 5.3% and 4.7 ± 3.1%, respectively). Similarly, an improvement in tricuspid regurgitation was noted, as 40% and 56% of patients showed absent or trivial PR at follow-up day 30 and after 1 year, respectively (Figure 2). Follow-up cardiac MRI conducted 1 year after the implantation revealed that the mean indexed RV end-diastolic volume had significantly decreased to 118.8 ± 8.9 mL/m^2^ (range: 110–138 mL/m^2^) after PPVI (*p* = 0.003; Figure 3; Table 4). In addition, the RV function (i.e., RV ejection fraction, which is determined by magnetic resonance imaging (MRI) showed a tendency to increase from 44.3% ± 11.6% (range: 20–63%) before PPVI to 51.3 ± 5.1% (range, 47–59%; Figure 4) at 1 year follow-up. Although these results are consistent with previous research showing no significant improvement in right ventricular ejection fraction (RVEF) after pulmonary valve replacement, RV systolic function turns out to improve significantly as a direct consequence of volume unloading after valve replacement especially when the RVEF was corrected for PR [19,20]. Moreover, the proportion of patients with NYHA class II (or higher) decreased from 85.7% at baseline to 26.2% at 30 days, and only two patients (26.2; 7.7%) showed NYHA class II after 6 months (Figure 5). Finally, both the VO_2_ and anaerobic threshold (AT) appeared to increase after PPVI; however, it should be noted that these variables were difficult to interpret given the small number of patients included in this study.

## 4. Discussion

In this study, we report the first experience of the utilization of both the balloon-expandable Melody valve and the self-expandable Pulsta valve for the treatment of RVOT dysfunction in Korea, in a relatively large sample population with an acceptable follow-up period. We successfully completed valve implantation in all patients without any device-related adverse events. TPV function was well maintained without any reintervention for a mean follow-up duration of 14.2 months.

Although the Melody valve was first implanted in a patient in the U.S. 13 years ago, reports regarding the use of the Melody valve have been lacking in many countries around the world, where the PPVI is still not routinely used and is in its introductory stage. Thus, this study is meaningful in showing the results of recent treatment outcomes that reflect advances in the latest technological experience.Indeed, the feasibility of the Pulsta valve for PPVI in Korea was first reported by Kim et al. in 2018 [14]. Since its approval for PPVI in Korea in 2019, the valve has been increasingly used. PPVI yields excellent early and short-term outcomes associated with a reduction in RVOT obstruction, elimination of PR, and clinical improvement of functional class. Specifically, improved circulatory efficiency and ventricular strain, enhanced cardiopulmonary exercise function in some patients, and better quality of life after PPVI have all been suggested [21,22,23,24].

Furthermore, several noteworthy changes in implantation practices and outcomes have been observed over time that likely reflect the learning curve effects, protocol modifications, and an evolving understanding of better practices [25,26,27,28]. Previously, patients were more likely to undergo PPVI with the smallest delivery systems (18 mm), which resulted in a high residual pressure gradient across the RVOT and more frequent stent fractures and reinterventions [6,7]. Furthermore, in 2007, Nordmeyer et al. reported a 21.1% (26 of 123) incidence rate of stent fractures after PPVI, which is main cause of TPV dysfunction and has become less common since pre-stenting became more widely implemented [18,25,29,30]. The indication of pre-stenting before PPVI with the Melody valve was to secure the landing zone on previous RVOT and to prevent valve frame fracture. The relief of the residual pressure gradient due to acute angulation or severe calcification on RVOT is also predominantly obtained by the pre-stenting also in our study. For the majority of our patients who underwent a valve-in-valve procedure, however, presenting was beneficial in case of resisting intrinsic or in situ recoil of the fractured previous frame rather than to prevent stent fracture. As a pre-stent occupies space within the previous bioprosthetic valve, potentially infringing on the achievable orifice area, pre-stenting in previous BPV and non-dynamic RVOT is even more controversial [31]. The largest 22 mm Melody delivery system was employed in all cases in our study without significant residual pressure gradient, while pre-stenting and/or the valve-in-valve procedure were performed in 29 (100%) patients. As a result, only one case of type 1 fracture was observed. The efforts made to prevent and reduce the occurrence of fractures of hemodynamically important valves are likely to yield even better long-term outcomes than those documented in previous studies [5,6,7,32].

Conduit rupture is a relatively common adverse event of PPVI, which occurs in around 19.5–22% of patients undergoing angioplasty [6,7]. As suggested in previous studies, we used a covered CP stent (CCPS; NuMED, Inc., Hoptkinton, NY, USA) in one patient with severe supravalvular stenosis and extensive calcification with luminal narrowing to prevent conduit injury and potential life-threatening ruptures. However, to prevent fatal coronary arterial compression, we performed selective coronary angiography with high-pressure balloon dilation to the maximal estimated diameter of the RVOT prior to TPV implantation in all cases. Indeed, previous reports suggested endocarditis to be an important adverse outcome during the follow-up period, however there is no endocarditis case reported in our study.

Intentional bioprosthetic valve frame fracture was attempted in eight (34.8%) patients to prevent a further decrease in the inner diameter (ID) that could potentially result in patient–prosthesis mismatch and to achieve larger ID with better hemodynamics after PPVI [31]. Either the 25 mm Saint Jude Biocor or the Epic valves had been previously used in seven patients, whereas the 23 mm Magna valve had been implanted in one subject. Among the patients with the bioprosthetic valve’s fracture, the median final ID was 1 mm larger (0.5–3 mm) than the valve’s true ID. Similarly, the inflation pressure required for the fracture varied between 18 and 20 atm (median 18 atm). Thereafter, Melody valves that were 22 mm in size were implanted in all subjects with a fractured frame, and no fracture-related adverse events were observed.

Compared to other self-expandable systems undergoing human clinical trials, the Pulsta valve has several unique advantages, including the knitted nitinol wire stent, which has a maximal length of 38 mm without bulkiness. Furthermore, the diameter of its outer sheath in the largest part of the TPV loading zone is only 18 F (5.9 mm). Therefore, the Pulsta valve is fit for various dynamic native RVOT lesions with significant PR given its low-profile delivery system and the softer nature of this device compared to other self-expandable pulmonic valves. Definitely, the Pulsta valve could be promising as a potential candidate for next-generation self-expandable valves for PPVI to minimize reintervention rates [14].

Although the implementation of PPVI started relatively late in Korea compared to that in other countries, considerable evidence has already accumulated through global experience. Encouragingly, the early and midterm outcomes of PPVI reported at our institution were remarkable and comparable to those in previous studies. In addition, in spite of the increasing use of these valves for non-conduit RVOTs, there are still patients whose RVOTs are too large to accommodate currently existing valve technology. Therefore, clinical trials on investigational devices, including the Venus *p*-valve, Harmony and Alterra Pre-stent (with SAPIEN S3) valves, have been carried out [33,34,35]. We suggest that the approval of the valves employed in our study, including the Pulsta valves, would greatly support the recovery of patients with an RVOT dysfunction and improve the outcomes of those with complex congenital heart diseases.

For one year of follow-up since 2019, in which the National Health Insurance reimbursement coverage of percutaneous pulmonary valve and implantation were applied, a total of 78 cases underwent successful valve insertion: 34 PPVI (34/78 = 43.6%) and 44 surgeries (44/78 = 56.4%). Overall, the reasons for surgery included those patients who needed primary and additional surgery (*n* = 20/78; 25.6%) due to tricuspid valvuloplasty, right ventricular outflow tract aneurysmectomy, or too small previous conduits considering patients’ growth, those where the RVOT was a priori too large for PPVI (>32 mm) (*n* = 18/78; 23.1%), and those with minor reasons (*n* = 6/78; 7.7%) including extensive acute endocarditis, coronary compression during balloon interrogation, and patients’ personal choice. As the Pulsta valve makes the procedure possible exceeding 28–29 mm of RVOT, a larger proportion of PPVI could be performed successfully compared to surgery than reported so far [36].

Considering the retrospective acquisition of the data, missing data had a negative impact on statistical significance. Furthermore, the small sample size included in the present investigation limited the reliability of the available variables. The ongoing collection of data at later time points will help to clarify longitudinal trends, and future studies with larger cohorts will augment to our findings. Nevertheless, given the early stage of PPVI in Korea, limited data on outcomes at later time points are available. More information will be obtained as the study continues with the follow-up of patients.

## 5. Conclusions

In conclusion, use of the Melody valve and the new self-expandable Pulsta valve for PPVI within the native RVOT has a good safety profile and high efficacy in terms of both hemodynamic and functional improvements. This appears to be comparable to previous reports with a potentially reduced complication rate. Nevertheless, the endurance of such positive safety and efficacy outcomes remains to be demonstrated over the longer term, as does device durability. This study showed, for the first time in Korea, the successful implementation of PPVI in patients with various sizes of RVOT. Continued data collection and initiation of larger registries in the future will add support for our findings.

## Figures and Tables

**Table 1:** Patient characteristics.

	*n*/N (%) or Mean ± SD (Range)
Age, years	27.2 ± 13.5 (9.5–75)
Female	12/42 (28.6)
Weight, kg	61.8 ± 14.3 (31–92)
Height, cm	173.8 ± 7.4 (124–185)
Prior endocarditis	3/42 (7.1)
Underlying diagnosis	
Tetralogy of Fallot	40/42 (95.2)
Pulmonary atresia with ventricular septal defect	2/42 (4.8)
RVOT anatomy prior to valve implantation	
Native RVOT	12/42 (28.6)
Bioprosthetic valve	24/42 (57.1)
Valved conduit	4/42 (9.5)
Homograft	2/42 (4.8)
Percutaneous pulmonary valve implant indication	
RVOT obstruction	15/42 (35.7)
Mixed PR and obstruction	14/42 (33.3)
PR	13/42 (31.0)
Cardiopulmonary exercise function	
Peak oxygen consumption, mL/kg/min	28.1 ± 4.7 (19.4–36.1)
Predicted peak oxygen consumption, %	57.4 ± 9.0 (36–73)
Oxygen consumption at the AT, mL/kg/min	8.0 ± 1.4 (5.5–10.3)
Maximum workload, W	11.1 ± 1.5 (7.4–12.4)

RVOT, right ventricular outflow tract; PR = pulmonary regurgitation; AT, anaerobic threshold; SD, standard deviation.

**Table 2:** Procedural characteristics.

	*n*/N (%) or Mean ± SD (Range)
General anesthesia	41/42 (97.6)
RVOT pre-stenting	10/42 (23.8)
Stent placed at the time of the procedure	9/10
Stent placed prior to the day of the procedure	1/10
Type of stent	
Palmaz 4014 Bare metal stent	8/10
CP covered stent	2/10
Size of the implanted Melody valve (*n* = 29)	
22 mm	29/29 (100)
Minimal diameter prior to pre-dilation	20.5 ± 2.9 (12–23)
Minimal diameter following pre-dilation	22.1 ± 0.6 (21.5–23)
Size of the implanted Pulsta valve (*n* = 13)	
26 mm	1/13 (7.7)
28 mm	3/13 (23.1)
30 mm	2/13 (15.4)
32 mm	7/13 (53.8)
Valve-in-valve (previously implanted bioprosthesis) procedure	28/42 (66.7)
Successful intentional BPV frame fracture	8/42 (19.0)
Length of procedure (min)	95.3 ± 35.8 (61–180)
Fluoroscopy time (min)	35.2 ± 10.1 (18.1–57.0)

RVOT, right ventricular outflow tract; CP, Cheatham Platinum; BPV, bioprosthetic valve; SD, standard deviation.

**Table 3:** Procedural outcomes.

		*n*/N (%) or Mean ± SD
Procedural success rate		42/42 (100.0)
Length of hospitalization, days		4.6 ± 0.7 (3–7)
Procedural complications		
Major	Valve dislocation	0 (0.0)
	Conduit/RVOT rupture requiring operation	0 (0.0)
Intermediate	Arrhythmia requiring treatment	0 (0.0)
	Coronary compression	0 (0.0)
	Stent dislocation	1 (2.4)
Minor	Contained conduit rupture	1 (2.4)
	Mild inguinal hematoma	1 (2.4)
Periprocedural and late mortality		1/42 (2.4)
Endocarditis		0/42 (0.0)
Stent fracture ^1^		1/42 (2.4)

RVOT, right ventricle outflow tract; SD, standard deviation. ^1^ Type 1, fracture of ≥ 1 strut without loss of stent integrity [18].

**Table 4:** Hemodynamic and functional improvements following percutaneous pulmonary valve implantation (PPVI).

		Baseline	Day 30	6 Months	1 Year	2 Years
Peak systolic gradient over the RVOT (mmHg)	Melody	51.3 ± 11.5	16.7 ± 3.3	16.5 ± 4.5	17.5 ± 5.8	23.0 ± 6.6
		(*n* = 29)	(*n* = 29)	(*n* = 22)	(*n* = 12)	(*n* = 8)
	Pulsta	14.2 ± 1.5	11.3 ± 1.2	10.0 ± 0.0	11.7 ± 1.2	
		(*n* = 13)	(*n* = 13)	(*n* = 4)	(*n* = 4)	
	*p*-value	<0.001	0.25	0.32	0.31	
RVSP across the tricuspid valve (mmHg)	Melody	70.0 ± 16.8	41.3 ± 17.8			
		(*n* = 29)	(*n* = 29)			
	Pulsta	41.8 ± 2.4 (*n* = 13)	39.0 ± 1.2 (*n* = 13)		40.7 ± 1.2 (*n* = 4)	
PRF(%) *	Melody	17.1 ± 13.6 (*n* = 26)			3.8 ± 5.3 (*n* = 12)	
	Pulsta	46.7 ± 8.0 (*n* = 13)			4.7 ± 3.1 (*n* = 4)	
	*p*-value	0.02			0.45	
RVEDVi (mL/m^2^) *	Melody	137.5 ± 31.1 (*n* = 26)			122.2 ± 8.3 (*n* = 12)	
	Pulsta	166.1 ± 11.9 (*n* = 13)			123.6 ± 12.4 (*n* = 4)	
	*p*-value	0.04			0.37	
RVEF (%) *		44.3±11.6 (*n* = 34)			51.3 ± 5.1 (*n* = 12)	
NYHA class						
Class I		6/42 (14.3)	31/42 (73.8)	19/23 (82.6)	14/16 (87.5)	8/8 (100.0)
Class II		24/42 (57.1)	11/42 (26.2)	4/23 (17.4)	2/16 (12.5)	0/8 (0.0)
Class III		11/42 (26.2)	0/42 (0.0)	0/26 (0.0)	0/16 (0.0)	0/8 (0.0)
Class IV		1/42 (2.4)	0/42 (0.0)	0/26 (0.0)	0/16 (0.0)	0/8 (0.0)
Peak oxygen consumption (mL O_2_/min/kg bodyweight)		28.1 ± 4.7 (*n* = 31)			31.8 ± 4.0 (*n* = 7)	
Anaerobic threshold (mL/min/kg)		8.0 ± 1.4 (*n* = 31)			9.1 ± 1.1 (*n* = 7)	

Values are presented as either the number/N (%) or mean ± standard deviation (range). PPVI, percutaneous pulmonary valve implantation; RVOT, right ventricular outflow tract; RVSP, right ventricular systolic pressure; PRF, pulmonary regurgitation fraction, RVEDVi, indexed right ventricular end-diastolic volume, RVEF, right ventricular ejection fraction; SD, standard deviation; NYHA, New York Heart Association. * Cardiac magnetic resonance image-derived measurements.
